# Supplementary material for: A two-step procedure to generate utilities for the Infant health-related Quality of life Instrument (IQI)
Source: PLoS One. 2020 Apr 3;15(4):e0230852. doi: 10.1371/journal.pone.0230852 (PMC7122817; doi:10.1371/journal.pone.0230852)
Supplement: S1 Table — (DOCX) [file pone.0230852.s005.docx]

**S3 Table**

Parameter estimates for the levels of the 7 IQI health items separately for the

general population and primary caregivers.

|  |  | General population (DC) | | |  | Primary caregivers (DC + Death) | | |  |
| --- | --- | --- | --- | --- | --- | --- | --- | --- | --- |
|  |  | Coefficient | SE | Significance |  | Coefficient | SE | Significance |  |
| Sleeping (2) |  | -.284 | .044 | .000 |  | -.151 | .038 | .000 |  |
| Sleeping (3) |  | -.360 | .048 | .000 |  | -.221 | .039 | .000 |  |
| Sleeping (4) |  | -.844 | .049 | .000 |  | -.522 | .039 | .000 |  |
| Feeding (2) |  | -.223 | .043 | .000 |  | -.137 | .037 | .000 |  |
| Feeding (3) |  | -.249 | .046 | .000 |  | -.058 | .038 | .128* |  |
| Feeding (4) |  | -.672 | .050 | .000 |  | -.395 | .038 | .000 |  |
| Breathing (2) |  | -.169 | .046 | .000 |  | -.202 | .038 | .000 |  |
| Breathing (3) |  | -.335 | .048 | .000 |  | -.317 | .039 | .000 |  |
| Breathing (4) |  | -1.001 | .051 | .000 |  | -.622 | .039 | .000 |  |
| Stooling (2) |  | -.014 | .042 | .738* |  | -.062 | .036 | .078* |  |
| Stooling (3) |  | .079 | .048 | .103* |  | .089 | .037 | .018 |  |
| Stooling (4) |  | -.262 | .058 | .000 |  | -.014 | .041 | .726* |  |
| Mood (2) |  | -.507 | .044 | .000 |  | -.305 | .037 | .000 |  |
| Mood (3) |  | -.418 | .045 | .000 |  | -.146 | .038 | .000 |  |
| Mood (4) |  | -.651 | .054 | .000 |  | -.250 | .040 | .000 |  |
| Skin (2) |  | -.142 | .042 | .000 |  | -.069 | .037 | .065* |  |
| Skin (3) |  | -.173 | .046 | .000 |  | .039 | .037 | .284* |  |
| Skin (4) |  | -.435 | .052 | .000 |  | -.137 | .038 | .000 |  |
| Interaction (1) |  | -.137 | .045 | .002 |  | -.154 | .037 | .000 |  |
| Interaction (3) |  | -.211 | .047 | .000 |  | -.192 | .039 | .000 |  |
| Interaction (4) |  | -.323 | .049 | .000 |  | -.265 | .040 | .000 |  |
| Death |  | N. A. | N. A. | N. A. |  | -2.401 | .081 | .000 |  |

***** Coefficients not significantly different than the baseline category (Level 1)

IQI = Infant Quality of life Instrument
