# Supplementary material for: A two-step procedure to generate utilities for the Infant health-related Quality of life Instrument (IQI)
Source: PLoS One. 2020 Apr 3;15(4):e0230852. doi: 10.1371/journal.pone.0230852 (PMC7122817; doi:10.1371/journal.pone.0230852)

**S4 Table**

Parameter estimates (values) for the levels of the
7 IQI health items for the primary caregivers, per country.


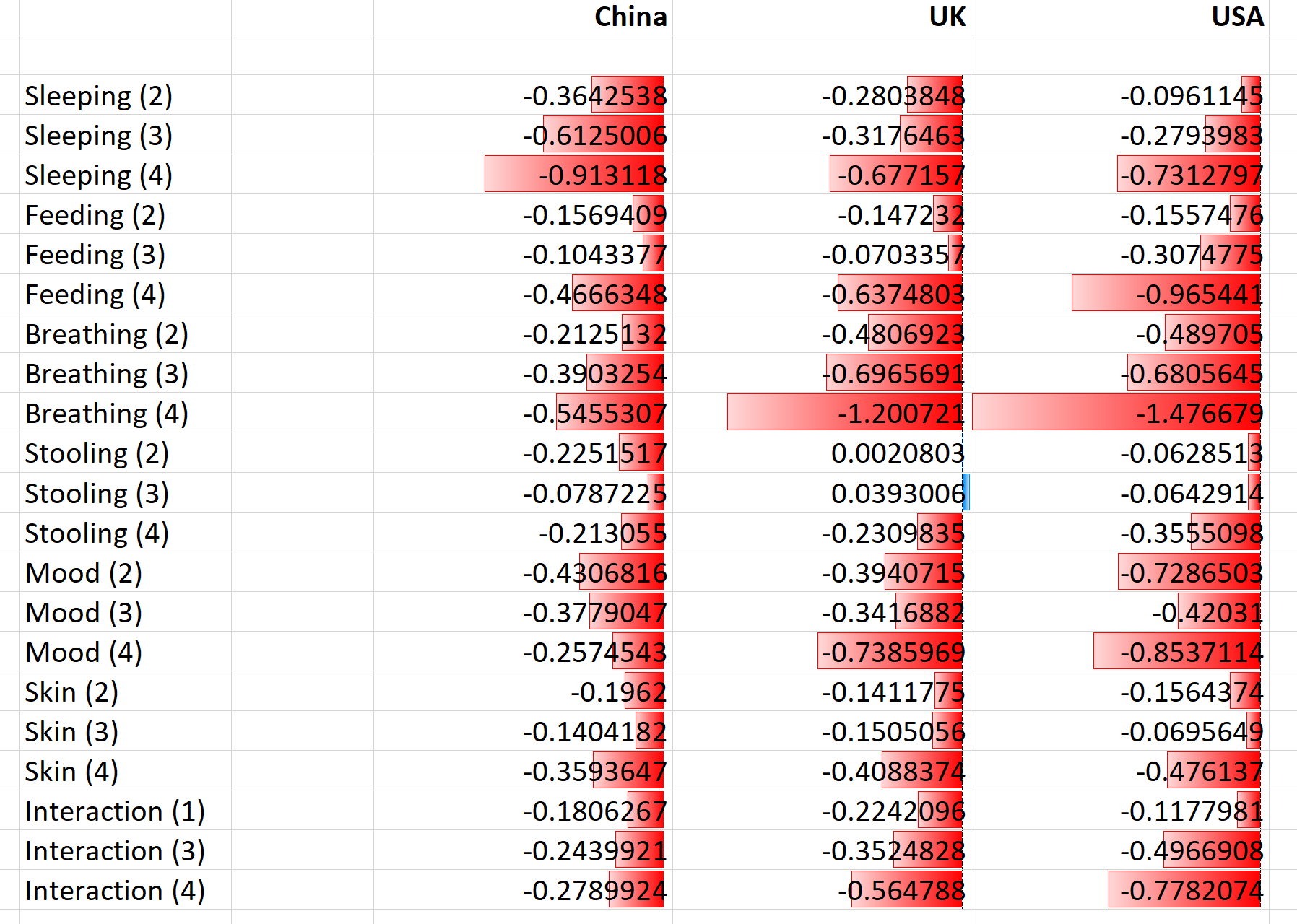

Supplement: S2 Table — (DOCX) [file pone.0230852.s006.docx]
