# Supplementary material for: A two-step procedure to generate utilities for the Infant health-related Quality of life Instrument (IQI)
Source: PLoS One. 2020 Apr 3;15(4):e0230852. doi: 10.1371/journal.pone.0230852 (PMC7122817; doi:10.1371/journal.pone.0230852)
Supplement: S3 Table — (DOCX) [file pone.0230852.s007.docx]

**S6 Table**

Parameter estimates (normalized scale = utilities) for the levels of the
7 IQI health items for the primary caregivers, per country.

|  |  | **China** | **UK** | **US** |
| --- | --- | --- | --- | --- |
|  |  | **Coefficient** | **Coefficient** | **Coefficient** |
|  |  |  |  |  |
| **Sleeping (2)** |  | **-.134** | **-.056** | **-.018** |
| **Sleeping (3)** |  | **-.226** | **-.063** | **-.051** |
| **Sleeping (4)** |  | **-.337** | **-.135** | **-.135** |
| **Feeding (2)** |  | **-.038** | **-.020** | **-.029** |
| **Feeding (3)** |  | **-.058** | **-.014** | **-.057** |
| **Feeding (4)** |  | **-.172** | **-.127** | **-.178** |
| **Breathing (2)** |  | **-.078** | **-.095** | **-.090** |
| **Breathing (3)** |  | **-.144** | **-.138** | **-.125** |
| **Breathing (4)** |  | **-.202** | **-.238** | **-.272** |
| **Stooling (2)** |  | **-.083** | **.000** | **-.012** |
| **Stooling (3)** |  | **-.029** | **.008** | **-.012** |
| **Stooling (4)** |  | **-.078** | **-.046** | **-.066** |
| **Mood (2)** |  | **-.159** | **-.078** | **-.134** |
| **Mood (3)** |  | **-.140** | **-.068** | **-.077** |
| **Mood (4)** |  | **-.095** | **-.147** | **-.157** |
| **Skin (2)** |  | **-.072** | **-.028** | **-.029** |
| **Skin (3)** |  | **-.052** | **-.029** | **-.013** |
| **Skin (4)** |  | **-.133** | **-.081** | **-.088** |
| **Interaction (1)** |  | **-.067** | **-.044** | **-.019** |
| **Interaction (3)** |  | **-.090** | **-.070** | **-.081** |
| **Interaction (4)** |  | **-.103** | **-.112** | **-.127** |
|  |  |  |  |  |
